# Narrativas de la conducta suicida en mujeres estudiantes de una universidad pública en México

**DOI:** 10.15446/rsap.V26n6.116184

**Published:** 2024-11-01

**Authors:** Nancy Araceli Méndez-Romero

**Affiliations:** 1 NM: Lic. Psc. Ph. D. Ciencias en Salud Colectiva. Cátedra en el Programa de Investigadoras e Investigadores del Consejo Mexiquense de Ciencia y Tecnología (COMECYT). Universidad Mexiquense del Bicentenario. Universidad Tecnológica de México, Campus Los Reyes. Estado de México, México. nmr2500@yahoo.com.mx Universidad Tecnológica de México Universidad Tecnológica de México México

**Keywords:** Suicidio, salud de las mujeres, género, universidad, violencia, desigualdad *(fuente: DeCS, BIREME)*, Suicide, women's health, gender, university, violence, inequality *(source: MeSH, NLM)*

## Abstract

**Introducción:**

La conducta suicida comprende diferentes fases: ideación, planeación e intento de suicidio. En México el suicidio representa la tercera y cuarta causa de muerte en mujeres de 10 a 14 y de 15 a 24 años, respectivamente.

**Objetivo:**

Analizar las experiencias de ideación e intento suicida de mujeres que cursan sus estudios en una universidad pública del estado de México (México).

**Métodos:**

Métodos Investigación cualitativa realizada de junio de 2023 a mayo de 2024. Se obtuvo una muestra intencional de cinco mujeres seleccionadas bajo determinados criterios: ser mayor de edad, tener un riesgo de suicidio de moderado a alto en la Escala de Riesgo Suicida Plutchik, haber recibido una orientación para el cuidado de la salud mental, estrategia utilizada para obtener las declaraciones personales sobre el comportamiento suicida. La información recolectada fue sistematizada en Atlas ti, se obtuvieron tres categorías las cuales fueron analizadas con enfoque de género en salud.

**Resultados:**

Se identificó que las participantes del estudio han intentado el suicidio alguna vez y que la ideación y el intento suicida ocurrieron desde etapas tempranas, lo cual les ha ocasionado un malestar psicoemocional persistente. Las experiencias sobre la conducta suicida están vinculadas a la exigencia de roles de género tradicionales, así como a vivir en entornos de violencia o carentes de apoyo.

**Conclusiones:**

La universidad requiere fortalecer actividades de acompañamiento académico y solidario, así como llevar a cabo acciones de prevención del suicidio, identificando condiciones de violencia u otras que afecten a mujeres estudiantes. Los casos en riesgo necesitan identificarse y derivarse a los servicios especializados.

## Abstract (en)

**Introduction:**

Suicidal behavior includes different phases: ideation, planning and suicide attempt. In Mexico, suicide represents the third and fourth cause of death in women aged 10 to 14 and 15 to 24, respectively.

**Objective:**

To analyze the experiences of suicidal ideation and attempts of women who are studying at a public university in the state of Mexico (Mexico).

**Methods:**

Qualitative research carried out from June 2023 to May 2024. An intentional sample of five women selected according to certain criteria was obtained: being of legal age, having a moderate to high risk of suicide on the Plutchik Suicide Risk Scale, having received orientation for mental health care, a strategy used to obtain personal statements about suicidal behavior. The information collected was systematized in Atlas ti, three categories were obtained which were analyzed with a gender focus on health.

**Results:**

It was identified that the study participants have attempted suicide at some time and that suicidal ideation and attempts occurred from early stages, causing persistent psychoemotional discomfort. The experiences of suicidal behavior are linked to the demand for traditional gender roles, as well as such as living in environments of violence and/or lack of support.

**Conclusions:**

The university needs to strengthen academic and solidarity support activities, as well as carry out suicide prevention actions, identifying conditions of violence or others that affect female students. Cases at risk need to be identified and referred to specialized services.

El suicidio es el acto deliberado de terminar con la propia vida 1. La conducta o comportamiento suicida, como se hará referencia, se expresa en distintas fases: ideación, planeación e intento suicida. Las muertes por esta causa difieren entre la población según el grupo de edad y sexo, por ejemplo, a nivel global el suicidio fue la cuarta causa de muerte en personas de 15 a 29 años 2. En México las personas de 18 a 29 años tienen una tasa de muerte de 10,7 por cada 100 000 habitantes. Al revisar los datos por sexo entre el 2017 y el 2021, el número de decesos de mujeres de 10 a 19 años pasó de 2,91 a 3,68 por cada 100 000 habitantes, mientras que en los hombres las tasas fueron de 11,04 y 14,15 por cada 100 000 habitantes 2.

Al delimitar esta problemática en mujeres, se identificó que la conducta suicida es la tercera causa de muerte en el grupo de 10 a 14 años y la cuarta en el grupo de 15 a 24 años 3. De acuerdo con Córdova-Osnaya 4 y Rubio-González 1, las mujeres presentaron notables niveles de ideación e intento suicida, los registros nacionales indicaron que la ideación tuvo una prevalencia del 5% en adolescentes de 12 a 17 años, en tanto que en el grupo de 18 a 49 años la prevalencia fue de 2,3%. Sobre el intento suicida, la prevalencia fue de 2,2% y 0,8% en el mismo grupo de edad 2. Los datos demostraron que las mujeres de 12 a 17 años presentan un riesgo significativo de ideación e intento de suicidio, por tanto, la detección y la prevención deben ser oportunas.

## La conducta suicida en las mujeres: antecedentes y abordajes

Entre los siglos XVII y XIX el discurso moral y religioso explicó la locura y el suicidio como un acontecimiento femenino, mientras que en el siglo XX el enfoque viró hacia el discurso médico, que ve al suicidio como una patología mental que afecta principalmente a las mujeres, con el argumento de que son más frágiles física y psíquicamente 5. Este enfoque, que abordó el suicidio como problema de salud, además estereotipó la conducta según el género: mientras que al hombre se le glorificó por decidir terminar con su sufrimiento mediante la muerte autoinfligida, a la mujer se le calificó como sufrible o cobarde 6.

El suicidio se ha abordado principalmente desde el campo biomédico-psicológico, aunque debido a su complejidad también se han considerado los aportes de la sociología y la antropología 5,6. De acuerdo con Pérez-Amezcua 7, los aspectos involucrados en la conducta suicida son:

a) individuales, como la depresión, el abuso de sustancias psicoactivas, los problemas de autoestima, entre otros; b) familiares, como la violencia de pareja, las presiones económicas, el antecedente de suicidio; c) comunitarios, como la violencia laboral o escolar; y d) estructurales, que abarcan el rol de género 8, el origen étnico 9,10 e incluso la disponibilidad de políticas, planes y programas de prevención del suicidio, incluyendo el acceso a servicios de salud mental.

Para ampliar la comprensión de la conducta suicida en las mujeres, a continuación se describen algunos estudios. En primer lugar, Acero Sánchez 6 en su investigación cualitativa con poblaciones indígenas de Chiapas (México) destacó la inexistencia de programas de acompañamiento a supervivientes de suicidio e identificó diferencias de género en su estigmatización; las mujeres recibieron mayores señalamientos por intentar morir. Por último, la autora refirió que las autolesiones en el cuerpo -cortes superficiales en brazos, piernas, quemaduras, golpes, entre otros- pueden ser una característica del comportamiento suicida en adolescentes, pues son señales que comunican el sufrimiento o la ruptura del sentido de la vida. Este hallazgo fue concurrente con Cardona-Arévalo 11, quien analizó las narrativas de autolesión en mujeres colombianas de 12 a 16 años, y encontró que las autoagresiones constituyen una vía de descarga y comunicación del malestar emocional.

Otras investigaciones cuantitativas se enfocaron en estudiantes de nivel medio superior y superior en México. En el nivel medio superior, el trabajo de Pérez-Amezcua 7, en el que participaron 12 424 jóvenes, identificó que el 47% de la muestra presentó ideación suicida y el 9% intento de suicidio. Los aspectos asociados a dicha ideación fueron: problemas de comunicación parental, abuso sexual, falta de acompañamiento emocional, ansiedad y escaso reconocimiento académico. En el intento suicida, además, se identificó el abuso de sustancias. Por otra parte, Cubilla-Rodríguez 12 en su investigación con 1 358 estudiantes de Sonora, reveló que las mujeres tuvieron mayor ideación e intento suicida. Finalmente, el estudio de Córdova-Osnaya 13, realizado en San Luis Potosí, demostró, mediante una muestra de 593 alumnos/as, que el 4,9 % presentó ideación suicida y que el trastorno emocional, la percepción de un futuro negativo, la exposición a situaciones adversas, así como la carencia de apoyo sociofamiliar incidieron en esta fase del comportamiento.

En el nivel superior, Córdova-Osnaya 4 observó que en una muestra de 584 alumnos/as de una universidad hidalguense, el 9,4% de los hombres tuvo ideación suicida y las mujeres la tuvieron en un 11,9%. La baja autoestima, el escaso apoyo familiar, las experiencias traumáticas, las bajas calificaciones y la mala condición económica se asociaron con la ideación en mujeres. La revisión de Rubio-González 1 también refirió que este grupo mostró mayor riesgo de comportamiento suicida y, al igual que Córdova Osnaya 4, reportó que la desesperanza, la hostilidad, la incomprensión, la soledad y los problemas familiares tienen relación con el problema. Por último, Granados Cosme 14 reportó una mayor prevalencia de esta conducta en estudiantes de medicina, comparado con alumnos/as de otras licenciaturas de una universidad pública en la Ciudad de México (CDMX); también reveló que los síntomas incrementaron conforme se avanzó en los estudios.

### Perspectiva de género en salud

La revisión de la literatura hasta ahora descrita delimitó el estudio de la conducta suicida como un problema de salud pública y para poder explicarla en el presente trabajo se utilizó la perspectiva de género en salud (PGS). Este abordaje reconoce que el proceso salud-enfermedad-atención-cuidado (S-E-A-C) se encuentra socialmente determinado por la condición socioeconómica, el origen étnico, el lugar de trabajo o residencia o el género; este último es una construcción que designa en las personas el cumplimiento de roles y estereotipos para definirse en los ámbitos de masculinidad o feminidad. Los cánones de género reproducen condiciones de desigualdad: mientras que los hombres cumplen con funciones de superioridad, trabajo productivo y remunerado, las mujeres realizan tareas que mantienen su subordinación u opresión, como, por ejemplo, el trabajo reproductivo y no remunerado que abarca el cuidado de otros. De acuerdo con Gómez-Gómez 15, la condición de género se sujeta a determinados perfiles de morbimortalidad, como se identificó en la ideación y el intento suicida en mujeres.

Gómez-Gómez 15 y la Organización Panamericana de la Salud (OPS), 16 indican que la incorporación de la PGS permite develar dimensiones de inequidad en este ámbito. Por ejemplo, las desigualdades de género se relacionan con los estados de enfermedad, en tanto que una segunda fuente de inequidad es el acceso a los servicios de atención sanitaria -educación y promoción de la salud, prevención de enfermedades, tratamiento o rehabilitación- 17. En el grupo de las mujeres se ha documentado que si bien ellas tienen una mayor esperanza de vida, también viven en continuos estados de enfermedad 16, lo que se debe a diversas causas de inequidad, como el estigma y la discriminación en el acceso a servicios médicos; una menor capacidad de pago que limita el acceso a la atención; la identificación tardía de patologías o el desconocimiento de sus necesidades de salud.

A partir de lo señalado en los anteriores acápites, se realizó el siguiente estudio para comprender la conducta suicida en un grupo de mujeres. Las preguntas de investigación fueron: ¿Qué aspectos individuales, familiares y/o socioculturales inciden en el comportamiento suicida de mujeres que cursan la universidad? y ¿Qué afectaciones viven las mujeres en la expresión de la conducta suicida? Por consiguiente, el objetivo fue analizar las experiencias de ideación e intento suicida de mujeres que realizan sus estudios en una universidad pública del estado de México, Edomex (México).

## MÉTODOS

Se trató de una investigación más amplia, llevada a cabo en diferentes etapas, con un enfoque mixto. Este documento presenta los resultados del análisis cualitativo.

### Selección y participantes

Se obtuvo una muestra intencional conformada por cinco mujeres elegidas de acuerdo con unos criterios específicos: ser estudiantes de licenciatura, tener entre 18 y 27 años, y haber obtenido un criterio de riesgo de suicidio al aplicarles la Escala de Riesgo Suicida Plutchik (ERSP) 18. El trabajo de campo se llevó a cabo entre junio de 2023 y mayo de 2024.

### Instrumentos y técnicas de recolección de información

Escala de Riesgo Suicida Plutchik 18, validada en población mexicana. El cuestionario tiene 15 reactivos, con respuestas de ausencia (equivalente a 0) y presencia (igual a 1). La obtención de un puntaje mayor a 6 se interpreta como riesgo suicida (RS). El RS son los determinantes personales, familiares o sociales que inciden en la expresión del suicidio en un momento determinado 1. También se aplicó un cuestionario de datos sociodemográficos.

Las técnicas cualitativas fueron: orientación para el cuidado de la salud mental (OCSM), hoja de notas, observación participante y diario de campo.

### Procedimiento

**Figura 1:**
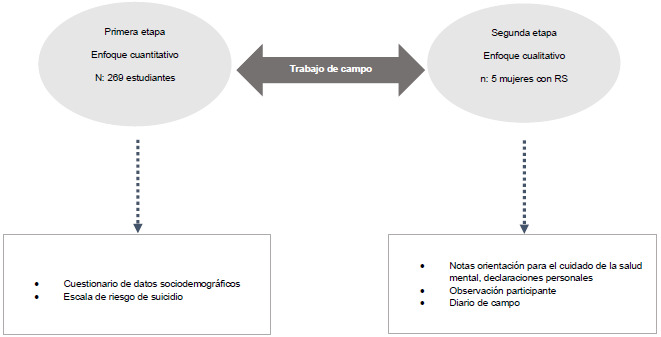
Etapas de investigación

La investigación se desarrolló en diferentes etapas, como se indica en la Figura 1, en la primera etapa se aplicó a estudiantes de una universidad pública del Edomex la cuidado de la salud mental (OCSM). De este grupo cinco ERSP. La segunda fase consistió en invitar a los alumnos/ mujeres autorizaron que su experiencia sobre la conducta as identificados con RS a recibir una orientación para el suicida fuera empleada con fines de investigación.

La investigadora fue quien brindó la OCSM, de forma virtual y presencial, en la universidad. Se programaron entre una y cuatro reuniones, con una duración de 30 minutos cada una, y se utilizó un abordaje terapéutico centrado en la solución para la prevención del suicidio 19. La OCSM tuvo como objetivo: explicar a las participantes los resultados de la ERSP; brindarles herramientas de afrontamiento al malestar psicoemocional y recopilar las declaraciones personales sobre la ideación y el intento suicida. De acuerdo con Schwartz y Jacobs 20, la técnica de declaraciones personales en investigación cualitativa se emplea para acceder a información que no es posible observar de forma directa.

### Procesamiento y análisis de la información

**Figura 2:**
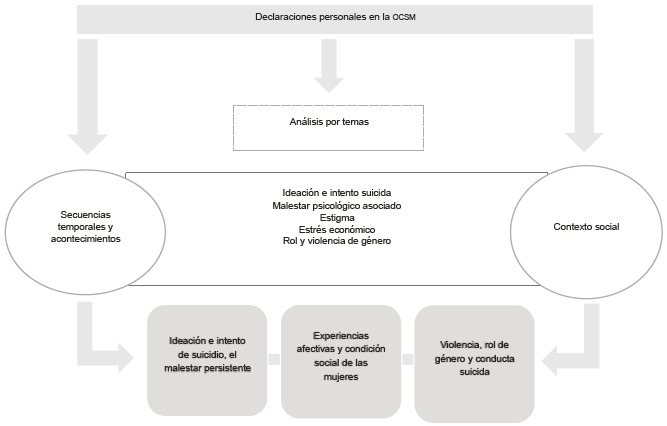
Procesamiento y análisis de la información cualitativa Declaraciones personales en la OCSM

Se recopilaron, se audiograbaron y se transcribieron en el programa Word, 11 sesiones de OCSM, luego de lo cual, los documentos se codificaron en el programa Atlas ti. Durante este proceso se identificaron las declaraciones personales y se elaboró el análisis por temas, considerando las secuencias temporales, el contexto social y el acontecimiento narrado 21. También se integraron las notas de OCSM y el diario de campo (Figura 2).

Se identificaron tres categorías emergentes: a) ideación e intento de suicidio, el malestar persistente; b) experiencias afectivas y condición social de las mujeres; c) violencia, rol de género y conducta suicida. Para la interpretación de los resultados se retomó la PGS.

### Criterios éticos

Se consideraron los principios de la Declaración de Helsinki para la investigación en seres humanos 22. El estudio contó con la firma del consentimiento informado, una vez que se explicó el objetivo, los beneficios y los riesgos mínimos, el uso de la información y la publicación de los hallazgos. La participación fue libre y voluntaria 23; se aseguró la confidencialidad y el anonimato. Los procedimientos con seres humanos fueron aprobados por la dirección académica de la institución donde se llevó a cabo el estudio.

## RESULTADOS

El rango de edad de las participantes fue de 18 a 27 años, tres son residentes del Edomex y dos de la CDMX. Una participante vive en pareja, las demás con la familia primaria; todas cursan una licenciatura del área de humanidades. Los resultados de la ERS evidenciaron que cuatro mujeres tuvieron un RS alto (mayor a 10 puntos), en tanto que una mostró un RS moderado (7 puntos). Las participantes han intentado el suicidio alguna vez, además, se autoperciben como ineficaces y reportan sensación de fracaso. Cuatro estudiantes consideran estar deprimidas, y de estas tres ven con desesperanza su futuro. Por último, dos jóvenes vivieron el suicidio de un familiar. Los hallazgos de las categorías emergentes se explican a continuación.

### Ideación e intento de suicidio, el malestar persistente

Este subapartado retoma a Gómez-Gómez 23, quien explica el intento suicida como un acontecimiento que da cuenta del mundo emocional de las mujeres. A partir de este hecho, se logra comprender las cualidades y las relaciones de su entorno social, el espacio donde se constituye la identidad femenina, donde se otorgan los significados a los hechos que marcan sus historias de vida. En otras palabras, el acto suicida y las emociones vinculadas que se identifican en las experiencias de las participantes develan la dimensión individual, así como su condición sociocultural.

A partir de los presupuestos anteriores, se analiza el relato de Alexandra, quien explica que días antes de recibir la sesión de OCSM había presentado ideación suicida (IS): "Quizá hace como unos 15 días [...] Los pensamientos que suelo llegar a tener son como de que, pues sencillamente ya no quiero saber cómo nada más de nada, ni de mí... Sencillamente yo digo no ¡pues ya mejor hasta aquí! pues ahora si como dicen ¡ahí muere!... Luego llego según yo, para calmarme, llego a subir a mi azotea y ahí me quedo pensando en ¡y si lo hago! y ¡si salto o si me aviento! "Entonces yo me he dado cuenta de que llega un punto de quiebre en el que pues sencillamente he considerado que lo mejor para mi es la salida fácil..." (Alexandra).

El relato de Alexandra describe sus pensamientos suicidas, así como el significado otorgado a la ideación suicida, que explica como un camino por seguir para desvincularse de las situaciones que vive. Al centrarse en el estado afectivo, se identifica su anhelo por experimentar tranquilidad. La situación de la participante es coincidente con Cristina, quien devela su intención por arrojarse al vacío, así como emplear el envenenamiento con medicamentos para finalizar experiencias emocionales percibidas como intolerables. Cristina lo narra de la siguiente manera: "Me dio como una crisis y ¡yo quería tomar muchas pastillas! o sea de todas las que encontrara. Y ha habido ocasiones en las que he pensado, así como que aventarme... desde arriba" (Cristina).

Otros de los hallazgos identifican las prácticas de autolesión en el cuerpo, lugar donde las mujeres descargan sus emociones, alcanzando la liberación momentánea del malestar psicoemocional. Esta conducta se expresa como un patrón de tensión-relajación 6. Desafortunadamente, las señales de autoagresión no se identificaron, ni tampoco recibieron la atención requerida. Por ejemplo, Paola describe sus prácticas autolesivas, así lo comparte: "Fue con una navaja, nada más fue como el rasguño. Mi cabeza se dio cuenta de que lo que volvía yo a hacer y pues...ya no lo seguí" (Paola).

Verónica, por su parte, describe las autolesiones y explica la forma en que esta acción se vincula con su intento de suicidio; estos acontecimientos tampoco fueron identificados por familiares; así lo narra: "Entrevistadora: ¿te ha pasado, así como que te da por..., a lo mejor cortarte las piernas? Verónica: Sí...lo llegue a hacer, tengo todas las marcas en los brazos y en las piernas tengo poquitos, eso fue parte de la secundaria. Fue con pastillas, de hecho, iba en la prepa… pero solo me pusieron a dormir y creo que… pues al siguiente día… mi mamá se enojó, porque me había despertado tarde y no había ido a la escuela" (Verónica).

Los relatos evidencian que la conducta suicida compromete el bienestar de quien la sufre, hallazgo que coincide con Gómez-Gómez 23. Al develar las experiencias de malestar emocional permanentes en las mujeres, los resultados demuestran que la autolesión expresada en el cuerpo, la ideación, el intento de suicidio se expresan desde la etapa adolescente y continúan hasta la juventud.

### Experiencias afectivas y condición social de las mujeres

El mundo emocional de las mujeres devela parte de la condición individual y relacional. Con referencia al segundo aspecto, se identificó que la ideación y el intento de suicidio ocurrieron en solitario, y también se evidenció la falta de apoyo de pares, familiares o personas cercanas en el contexto escolar, dejando a las mujeres sentimientos de soledad, tristeza, temor al rechazo y desesperanza. En el caso de Cristina, la falta de protección y la indiferencia han marcado su experiencia; así lo comparte: "Hay veces en las que yo me siento muy sola, yo no me siento, así como que apoyada por alguien e incluso amigos, yo nunca les he dicho, así como que tengo problemas y así, ¡nada más yo lo sé y nadie más. Por ejemplo, yo perdí a mi abuelito, a mi novio y a mi mejor amiga, se fueron y fue como que yo me quedé, así como que, sin nada ¡muy triste".

Con respecto a la situación emocional vinculada con los intentos de suicidio, se reconoció la minimización de los estados afectivos femeninos o, en el peor de los casos, las mujeres fueron juzgadas por experimentar temor ante las condiciones de vida, lo que indica que de ellas se espera autosuficiencia para solucionar sus problemas y continuar con los deberes. La historia de Cristina devela que, además de la tristeza, ella fue cuestionada por expresar su deseo de pedir ayuda especializada; la joven refiere el desinterés expresado por sus relaciones más cercanas: "He tenido ganas de pedirle ayuda a mis papás, pero… mi mamá es así como que, luego tantito luego le digo algo y me dice ¡ay en mis tiempos nada de que psicólogo, te pegaban una chinga" (Cristina).

Alexandra, otra de las mujeres, narra su experiencia emocional debido a la falta de acompañamiento, en la que se evidencia indiferencia hacia su persona; el temor, así lo expresa: "Siento que se me juntan varios factores, con mi pareja, conmigo misma y con mi familia, que a lo mejor yo quiero hacer algo, pero ¡me da miedo! Y a la vez me entristece el hecho de saber que a lo mejor las personas con las que yo creía o creo contar más a veces son las personas de las que… más me atacan, eso me hace sentir bastante mal porque, pues porque a la vez ¡me siento sola" (Alexandra).

La desesperanza hacia el futuro es otra experiencia afectiva vivida, que se genera por la falta de reconocimiento de los logros personales, las cualidades individuales y las capacidades de las mujeres. La falta de interés por parte del grupo familiar y escolar hacia lo que les sucede a las mujeres influye en la construcción de una identidad basada en la desconfianza hacia ellas, lo que reafirma el temor y el desamparo que sienten ellas ante los desafíos que enfrentan, como los académicos. Cristina narra así su experiencia: "Luego yo también digo o sea porque nos esforzamos tanto, digo ¡todos algún día nos vamos a morir!, son pensamientos así que me vienen de la nada" (Cristina).

Del mismo modo, Verónica ha vivido la indiferencia en el entorno escolar, pues en una ocasión al expresar lo que estaba viviendo no recibió acompañamiento emocional, y tampoco se le brindaron alternativas para apoyar su situación académica, lo que hizo aumentar su estado de preocupación: "Un maestro me dijo: ¡Verónica! ¿Qué está pasando contigo? y yo todavía sarcásticamente le dije: ¿por qué? Sí, ¡los profesores ya se están quejando que no entras a las clases, te van a mandar a extraordinario! y yo le dije ¡ay no".

Lo destacable en estos relatos fue constatar que la ideación y el intento suicida no se reconocieron, ni tampoco se confirmó la persistencia de la conducta suicida, ya que en el ámbito universitario el malestar psicológico continuó expresándose. Los hechos narrados develaron la condición y el escaso acompañamiento hacia las estudiantes, pues incluso en la universidad su situación fue interpretada como falta de compromiso académico, lo que refuerza la percepción de ineficacia en su formación profesional.

### Violencia, rol de género y conducta suicida

El análisis pone en evidencia que las situaciones familiares y el rol de género inciden en el comportamiento suicida. En el ámbito familiar, el relato de Verónica muestra la violencia en el hogar. Las situaciones vivenciadas en este espacio de sociabilidad, lejos de representar un lugar de seguridad para ella, se conforman como una fuente de ansiedad, estrés e incertidumbre.

De acuerdo con la experiencia de la participante, el antagonismo entre los padres afectó su trayectoria académica. Así, recordó que en una ocasión presenció las disputas entre sus progenitores y ello le causó distracción para contestar su examen de ingreso a la universidad. Finalmente, el entorno violento en la familia modificó sus planes personales y académicos, lo que afectó su bienestar al continuar recibiendo los reclamos, la culpabilización, el fracaso y la falta de soporte emocional y escolar. Verónica lo narra de la siguiente manera: "Mi mamá lo corrió [al padre]. todas las noches se la pasaban discutiendo y así y ya para el día de irme a hacer el examen, sí me daba miedo de que le fuera hacer algo a ella, hasta que le fuera a pegar algo así, entonces al momento de hacer mi examen, pues traté de calmarme, pero no se pudo. Al momento de que salen los resultados… pues si me puse mal y todo eso, y hasta ella me regañó, ¿Por qué no te quedaste? [en la universidad] Me hizo enojar que yo le dije que, si ella no se hubiera peleado con la pareja, un día antes. Se regresó [la madre de la joven] se enojó y me dijo ¡pues si tanto te molesta, si tanto te enojas o me culpas, de que no quedaste en la universidad pues está bien, yo me voy de la casa! Yo ni siquiera mi casa la siento como un lugar seguro, ni siquiera siento que sea mi casa...".

El siguiente relato también muestra la violencia que el padre ha ejercido sobre la madre. Lamentablemente, en la historia familiar de Cristina ello se ha asumido como una forma de convivencia, lo que refuerza el rol de fragilidad y dependencia emocional de las mujeres hacia el autoritarismo masculino. En el caso de esta participante, el abuso le ocasionó sentimientos de culpa e indefensión. En el entorno social, los familiares incorporaron las violencias como un asunto privado, restringiendo la búsqueda de alternativas institucionales: "Toda mi familia, la de mi mamá y la de mi papá, se han enterado de los problemas que han tenido mis papás, incluso una vez mi abuelita nos dijo: ¿Qué por qué querían demandar a mi papá? y este mi abuelito nos estaba diciendo que era lo que teníamos que decir […] Pero pues a mí me ponían entre la espada y la pared porque yo quiero a mis dos papás, yo tampoco quería que a mi papá lo metieran a la cárcel... al final de cuentas pues ya se reconciliaron y ahorita siguen juntos" (Cristina).

En el caso de Viviana, ella recuerda que desde su niñez su padre las agredía a ella y a su madre. Las violencias vividas son el sometimiento al autoritarismo, el castigo y la descalificación; las expresiones de las que es objeto la joven son el maltrato físico, psicológico y económico. Además, las experiencias de la participante evidencian los imperativos de género por cumplir; las demandas son: obediencia; identificarse como una fuente de estrés económico para la mamá; cumplir con dobles cargas de trabajo, como estudiar y realizar tareas del hogar; este último aspecto confirma que en el seno familiar se exige el cumplimiento del rol tradicional femenino: "Me defendí, me intentó pegar [el padre], me jaló de las manos, mi tío y mi abuelita vieron, pensé: ¡una ayudadita!, me comenzó a pegar, me dio de cinturonazos. [Su padre le dice] ¡No sabes cocinar!, si no cocino bien mi papá me tira la comida y cuando mi papá se enoja no da dinero, le da más carga económica a mi mamá (Viviana)".

El relato de Cristina, al igual que el de Viviana, revela la vulnerabilidad que sienten las mujeres. Además, los abusos cometidos con ellas por ser mujeres muestran el entorno de inseguridad en el que viven. La reproducción y el silenciamiento de eventos violentos, el temor, el desamparo, la falta de alternativas, la desprotección física y emocional desde la infancia han caracterizado a las historias de estas jóvenes. El relato de Cristina así lo comprueba: "Yo tengo un recuerdo de cuando mi mamá iba a trabajar […] mi mamá iba a trabajar de limpieza y yo me acuerdo de que me metía a un baño y también había otro señor que trabajaba de limpieza, y yo me metí al baño y yo me acuerdo de que el señor se metió, me acuerdo de que estaba sentadita en el baño, y el señor se metió y yo le dije que estaba ocupado y el señor no le importó… me acuerdo de que estaba haciendo del baño y el señor estaba ahí...".

Finalmente, se identificó que la violencia contra las mujeres está arraigada en el entorno familiar y se reproduce en los demás ámbitos, al establecerse relaciones de subordinación, opresión y violencia, así como desinterés hacia la igualdad y el bienestar de las mujeres. Las condiciones de género de las participantes se expresaron en sus estados afectivos, entre los cuales la idea de muerte o el intento suicida se perciben como una alternativa para alejarse de estas formas de abuso y relación familiar, en busca del anhelo de liberarse con la muerte de los daños sufridos 23.

## DISCUSIÓN

El objetivo fue analizar las experiencias de ideación e intento suicida de cinco mujeres. Los resultados fueron: todas tuvieron un intento de suicidio en su hogar; se confirmó la autolesión como señal de alerta; planear o aventarse al vacío o el envenenamiento con medicamentos fueron los mecanismos empleados. A nivel nacional, estos métodos son utilizados con frecuencia por adolescentes 2.

También se identificó la persistencia del malestar psicoemocional y que el comportamiento suicida suele manifestarse en etapas tempranas de la vida, pese a ser prevenible 1,7,12,18. Los resultados, junto con los hallazgos de Gómez-Gómez 23, confirman el contínuum del intento suicida en las mujeres. Además, según el autor, este problema se vincula con historias marcadas por relaciones de violencia emocional, dependencia e inequidad que evidencian el retraso del apoyo sociofamiliar o institucional. En este orden, los resultados de la investigación develaron las violencias y las desigualdades de género, en coincidencia con los estudios realizados con adolescentes 7 jóvenes 1,4 y mujeres adultas 24. Paralelamente, se evidenció que la conducta suicida, además de ser una afectación individual, daña a mujeres que comparten condiciones socioculturales similares.

Otro hallazgo fue la expresión de la conducta suicida en escenarios carentes de apoyo 4, desinterés, distancia-miento afectivo 1, problemas económicos, desesperanza hacia el futuro y estigma 6. Tampoco hubo acompañamiento académico 14. En este sentido, respecto Cardona-Arévalo 11 sugiere que las instancias académicas, al ser receptoras de estas problemáticas, requieren implementar actividades promotoras de salud y bienestar para los estudiantes.

Por último, si bien los resultados no pretenden generalizarse, es posible reconocer situaciones de otras jóvenes en condiciones similares a las de las mujeres del estudio. En consecuencia, se recomienda identificar el malestar psicoemocional durante etapas tempranas de vida; atender las violencias de género; garantizar el acceso a las acciones de prevención del suicidio en el ámbito escolar 24; reducir el estigma, promoviendo el apoyo colectivo, pues, como lo menciona Valdez-Santiago 2, la prevención del suicidio en las juventudes debe ser prioritaria en la agenda nacional de salud. Finalmente, queda abierta una línea de investigación en varones y personas de la diversidad sexo-genérica, quienes también presentan indicadores de morbimortalidad por esta causa ♦
